# Female reproductive health impacts of Long COVID and associated illnesses including ME/CFS, POTS, and connective tissue disorders: a literature review

**DOI:** 10.3389/fresc.2023.1122673

**Published:** 2023-04-28

**Authors:** Beth Pollack, Emelia von Saltza, Lisa McCorkell, Lucia Santos, Ashley Hultman, Alison K. Cohen, Letícia Soares

**Affiliations:** ^1^ Department of Biological Engineering, Massachusetts Institute of Technology, Cambridge, MA, United States; ^2^ Patient-Led Research Collaborative, Washington, DC, United States; ^3^ Department of Epidemiology & Biostatistics, School of Medicine, University of California, San Francisco, San Francisco, CA, United States

**Keywords:** Long COVID, reproductive health, myalgic encephalomyelitis, endometriosis, postural orthostatic tachycardia syndrome, Ehlers-Danlos sydrome, post-acute sequalae of SARS-CoV-2 infection, female

## Abstract

Long COVID disproportionately affects premenopausal women, but relatively few studies have examined Long COVID's impact on female reproductive health. We conduct a review of the literature documenting the female reproductive health impacts of Long COVID which may include disruptions to the menstrual cycle, gonadal function, ovarian sufficiency, menopause, and fertility, as well as symptom exacerbation around menstruation. Given limited research, we also review the reproductive health impacts of overlapping and associated illnesses including myalgic encephalomyelitis/chronic fatigue syndrome (ME/CFS), postural orthostatic tachycardia syndrome (POTS), connective tissue disorders like Ehlers-Danlos syndrome (EDS), and endometriosis, as these illnesses may help to elucidate reproductive health conditions in Long COVID. These associated illnesses, whose patients are 70%–80% women, have increased rates of dysmenorrhea, amenorrhea, oligomenorrhea, dyspareunia, endometriosis, infertility, vulvodynia, intermenstrual bleeding, ovarian cysts, uterine fibroids and bleeding, pelvic congestion syndrome, gynecological surgeries, and adverse pregnancy complications such as preeclampsia, maternal mortality, and premature birth. Additionally, in Long COVID and associated illnesses, symptoms can be impacted by the menstrual cycle, pregnancy, and menopause. We propose priorities for future research and reproductive healthcare in Long COVID based on a review of the literature. These include screening Long COVID patients for comorbid and associated conditions; studying the impacts of the menstrual cycle, pregnancy, and menopause on symptoms and illness progression; uncovering the role of sex differences and sex hormones in Long COVID and associated illnesses; and addressing historical research and healthcare inequities that have contributed to detrimental knowledge gaps for this patient population.

## 1. Introduction

Long COVID (LC) is a disabling illness that can develop in anyone after a SARS-CoV-2 infection, independent of severity of COVID-19 disease. LC is defined as experiencing symptoms within three months from the initial infection that last at least two months (1). Symptoms can include fatigue, cognitive dysfunction, post-exertional malaise (PEM), headaches, insomnia, and muscle aches (2, 3). LC pathophysiology includes immune dysregulation and autoimmunity, pathogen persistence/reactivation, neurological abnormalities and neuroinflammation, tissue and organ damage, hypoperfusion and autonomic dysfunction, fibrin amyloid microclots, and microbiome dysregulation (3–7).

Evidence suggests that LC affects twice as many women as men (8) and may disproportionately impact transgender people (9). Premenopausal women have an elevated risk for LC (8), suggesting that sex hormones may play a key role in LC development (10)*.* Reproductive health (RH) conditions are common pathologies within LC, but they remain significantly understudied.

This review examines evidence of female RH symptoms among LC patients. Given limited research, we also review RH impacts of associated illnesses including myalgic encephalomyelitis/chronic fatigue syndrome (ME/CFS), postural orthostatic tachycardia syndrome (POTS), connective tissue disorders like Ehlers-Danlos syndrome (EDS), and endometriosis. Like LC, these illnesses predominantly impact women (11–13) and may help elucidate reproductive health in LC. This review highlights the importance of studying RH in LC, especially as RH conditions in general have been historically under-researched (14–16). It also proposes research priorities to advance knowledge of RH in LC and to improve LC patient outcomes.

## 2. Female reproductive health in Long COVID

Few studies have investigated the impact of LC on female RH, and emerging research suggests that LC can impact the menstrual cycle, ovarian health, and fertility.

### 2.1. Long COVID and the menstrual cycle

Research has found that premenopausal women with LC may commonly experience worsening of premenstrual symptoms and/or exacerbation of LC symptoms linked to menstrual cycle changes. In one cross-sectional study (*n* = 1,792), over one-third of menstruating LC patients reported an exacerbation of symptoms the week before or during menses (2). In another cross-sectional study (*n* = 460), 62% of LC patients experienced symptom worsening on days prior to menses (17).

LC patients report experiencing menstrual cycle irregularities, including changes to the length of the cycle, duration, and intensity of the menses. In a multi-country patient-led survey of 1,792 LC patient respondents with a menstrual cycle, 33.8% reported menstrual issues, which included abnormally irregular cycles (26%) and heavy periods (19.7%) (2). Additionally, 4.5% of 1,123 cisgender women aged 49 or older reported post-menopausal bleeding (2). Another survey study found that LC patients (*n* = 748) report higher rates of menstrual cycle changes (OR 1.34, 95% CI 1.15–1.57) compared to the general population, both with (*n* = 2,299) and without (*n* = 15,156) a history of COVID-19 (OR 0.99, 95% CI 0.91–1.09) (18). A longitudinal prospective cohort study with no control arm found that 16% of women and nonbinary people experienced menstrual cycle changes 28 to 222 days after SARS-CoV-2 infection (19). These include irregular or infrequent menstruation and increased premenstrual syndrome symptoms. A retrospective case-control study comparing the effects of COVID-19 (*n* = 1,066) and vaccination (*n* = 4,989) on menstrual health found that a history of COVID-19, but not vaccination, was associated with an increased risk of changes in menstrual cycle duration, bleeding between periods, increased menstrual flow, and missed periods (20).

Limitations of most of these studies include lack of healthy control or comparison groups, not screening participants for associated illnesses or comparison with these illnesses, and lack of consideration in the study design of factors that may impact menstrual cycles, such as vaccination (21).

### 2.2. Long COVID, fertility, and ovarian health

Case reports suggest that COVID-19 infection may be associated with long-term decline of ovarian health, including premature ovarian insufficiency (POI), a loss of normal ovary function before age 40. Based on transvaginal ultrasound examination and follicle-stimulating hormone (FSH) testing, POI was reported in a 34-year-old patient who had been experiencing irregular menstrual cycles and abnormally low bleeding since COVID-19 infection 12 months prior (22). Seven months after COVID-19, another 34-year-old LC patient was diagnosed with POI due to elevated gonadotropin levels and irregular menstrual cycles with oligomenorrhea (23). The patient's menstrual cycle was regular and hormone levels were normal two months before having COVID-19 (23). Eight months post-COVID-19, a 27-year old patient, who had been experiencing amenorrhea since infection, was diagnosed with POI based on high levels of gonadotropin hormones (FSH and LH) and menopausal levels of estradiol (24). Patients in these case reports were not screened for LC associated illnesses (e.g., ME/CFS or POTS), hampering the understanding of the pathophysiology of POI in LC. Given that only case reports exist, there are no estimates of the post-COVID-19 incidence of POI or its relation with associated illnesses, and whether it is more prevalent among people with LC.

Follicular fluid composition may be altered months after COVID-19 infection, which could affect oocyte quality and overall fertility (25). Levels of the cytokine IL-1 and vascular endothelial growth factors (VEGF) — key peptides in angiogenesis and vascular permeability and thus, oocyte development — were lower in the follicular fluid of people undergoing assisted reproductive treatment 2 to 9 months (average 4.5 months, *n* = 46) post-COVID-19 compared to controls who were SARS-CoV-2-negative or never had COVID-19 symptoms (*n* = 34) (26).

Premature menopause, associated with long-term health risks including increased morbidity and mortality (27), was reported by 3% of a sample of 938 cisgender women with LC in their 40s (2) who had persistent symptoms for 7 months post-COVID-19. This finding is higher than pre-pandemic estimates of premature menopause among cisgender women in their 40s [e.g., 1% in the US (28)].

### 2.3. Long COVID and pregnancy

Few studies have investigated LC in pregnant people. A cross sectional survey in Ecuador found that pregnant (*n* = 16) and non-pregnant (*n* = 231) women with LC experienced the same symptoms, with the three most commonly reported symptoms for both groups being fatigue, hair loss, and difficulty concentrating (29). A US prospective cohort study of pregnant people found that 25% had LC symptoms eight or more weeks after testing positive for SARS-CoV-2 (30). However, studies have not yet examined how LC affects pregnancy.

A small but important control-matched prospective cohort study in Brazil (*n* = 88) followed pregnant women after testing positive for COVID-19 (*n* = 84), finding that 75.9% developed LC (31). This study also found that patients given glucocorticoids to treat COVID-19 during pregnancy were at higher risk (RR 6.92, 95% CI 1.70–28.07) of persistent fatigue (31), a key and debilitating LC symptom.

## 3. Female reproductive health in illnesses comorbid or associated with Long COVID: A brief overview of myalgic encephalomyelitis/chronic fatigue syndrome (ME/CFS), postural orthostatic tachycardia syndrome (POTS), Ehlers-Danlos syndrome (EDS), and endometriosis

**Figure 1:**
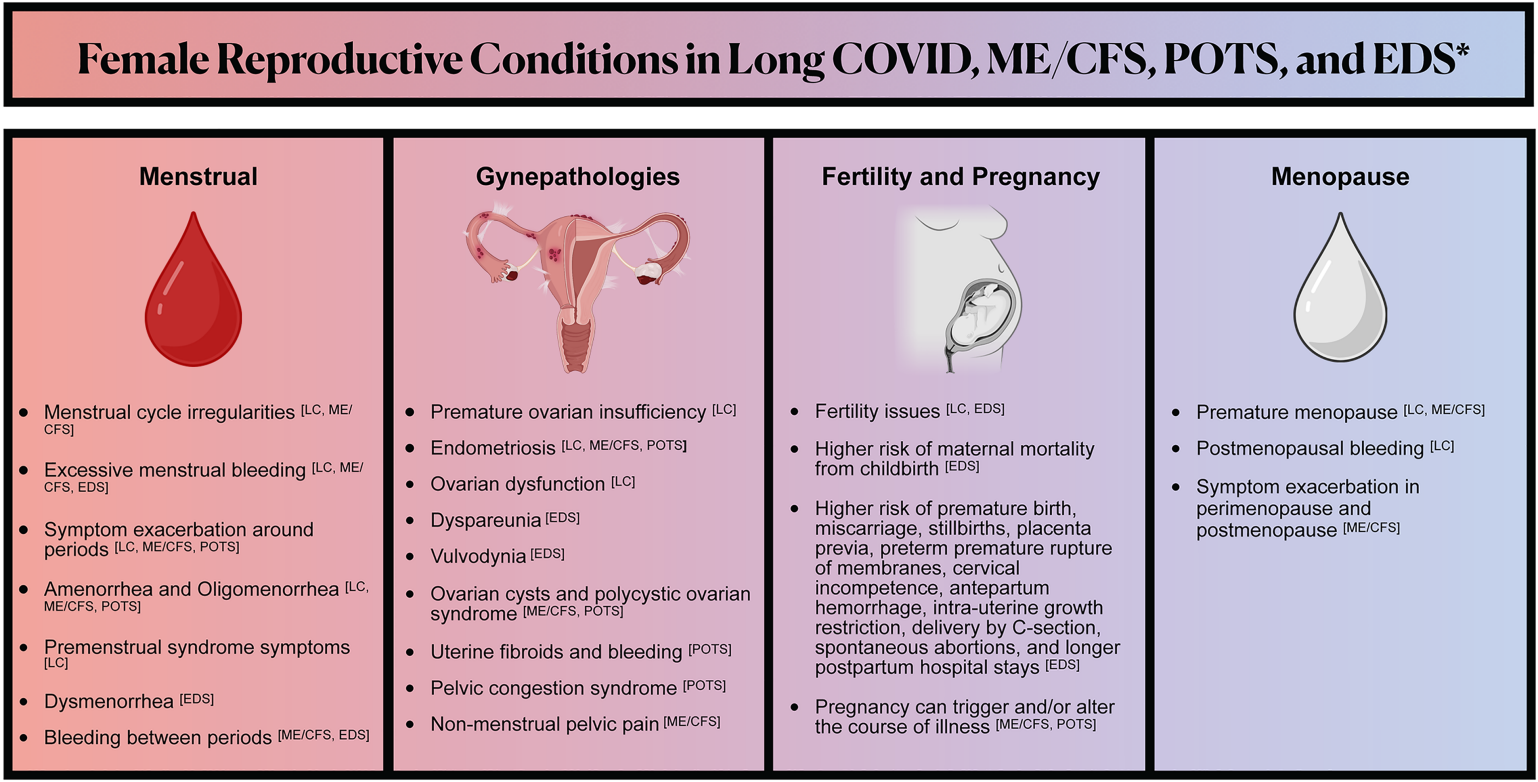
Illustrates the reproductive symptoms and conditions that may be associated with Long COVID, myalgic encephalomyelitis/chronic fatigue syndrome (ME/CFS), postural orthostatic tachycardia syndrome (POTS), and Ehlers-Danlos Syndrome (EDS). Reproductive symptoms and conditions in illnesses associated with Long COVID are highlighted to help elucidate knowledge gaps in Long COVID reproductive health. ^*^The lists of symptoms and conditions represented are non-exhaustive and reflect what is discussed in this literature review. There are relevant gaps in reproductive health research in all of the conditions represented.

Given minimal research on RH conditions in LC, we draw upon the limited, often cross-sectional research on RH in ME/CFS and POTS — illnesses frequently triggered by infection that many people with LC develop (4, 5, 32–35). Additionally, we examine research on connective tissue disorders, namely EDS, and endometriosis, illnesses with significant RH implications that are comorbid with ME/CFS and POTS and may be associated with LC. See Figure 1 for a summary of the female reproductive conditions we review in Long COVID, ME/CFS, POTS, and EDS.

### 3.1. Myalgic encephalomyelitis/chronic fatigue syndrome (ME/CFS) and reproductive health

Approximately 45% of LC patients develop ME/CFS (33, 34), a multi-system neuroimmune illness characterized by: neurological, vascular, and cognitive symptoms; pain; extreme fatigue unrelieved by sleep; and an exacerbation of symptoms or “crash” after physical or cognitive exertion (PEM) (33, 36). Infection is the most common onset event for ME/CFS (37), accounting for up to 75% of cases (5).

Female sex is a significant and consistent risk factor for ME/CFS (38), and sex and endocrine events influence the course of the illness (38). Many women with ME/CFS report that menstrual cycles, pregnancy, and menopause exacerbate symptoms (37). Compared to healthy controls, women with ME/CFS have disproportionately reported irregular menstrual cycles (39), amenorrhea (39, 40), excessive menstrual bleeding (41), bleeding between periods (39, 41), non-menstrual pelvic pain (40, 41), endometriosis (40, 42), gynecological surgery (especially hysterectomy) (41), and a history of polycystic ovarian syndrome (PCOS) and ovarian cysts (39). Half to two thirds (53%–67%, *n* = 120 and *n* = 42) of female ME/CFS patients report increased symptoms before menstruation, though these survey-based studies lack healthy controls (37, 43). A longitudinal case-controlled study (*n* = 157) revealed that early onset menopause is a risk factor for ME/CFS; mean age of menopause in ME/CFS (37.6 y) was earlier than healthy controls (48.6 y) (41), possibly due in part to gynecological surgery (40). Menopause exacerbated symptoms in 38% (*n* = 150) of perimenopausal and postmenopausal women with ME/CFS in a cross-sectional survey study (37).

Pregnancy is reported as a trigger for ME/CFS in 3%–10% of cases (4, 37). A study (*n* = 77) with age, sex, and education matched controls found that women who had been pregnant in the previous year were over 31 times more likely to develop ME/CFS (37, 44). Evidence is mixed for symptomatology among ME/CFS patients who become pregnant: nearly equal subsets may see symptoms improve, stay the same, or worsen (37).

### 3.2. Postural orthostatic tachycardia syndrome (POTS) and reproductive health

POTS is a type of dysautonomia involving orthostatic tachycardia without orthostatic hypotension (35). Common symptoms include lightheadedness, tachycardia, presyncope, and headaches (35). Among LC patients, 28% and 30% (*n* = 42, *n* = 70) had POTS in two NASA lean test and active stand test studies (32, 45). Pre-pandemic prevalence of POTS is estimated at 0.2%–1% (46), with 41% of POTS (*n* = 1,933) patients citing an infectious trigger in a non-controlled survey study (35).

Menstruation can significantly impact POTS symptoms. Menstrual cycle hormones have been found to affect hemodynamics (cardiac output, stroke volume, and total peripheral resistance) in POTS patients but not in healthy controls, possibly by modulating the renin-angiotensin-aldosterone system (47). Elevated estrogen and progesterone in the mid-luteal phases may increase blood volume retention and are associated with increased levels of renal-adrenal hormones (47). A prospective, questionnaire-based study of POTS patients (*n* = 65) and healthy controls (*n* = 95) found that dizziness fluctuated in the menstrual cycle, with greatest lightheadedness during menses, a decrease in the follicular phase, lowest in the mid-luteal phase, and an increase in the late luteal phase (48).

Studies (*n* = 65 and *n* = 191) have found increased rates of dysfunctional uterine bleeding, secondary amenorrhea, uterine fibroids (25% vs. 10% of healthy controls), endometriosis, ovarian cysts (43% vs. 13% of healthy controls), and pelvic congestion syndrome in POTS patients (48, 49). A cross-sectional survey study of 3,652 POTS patients reporting at least one pregnancy found that 81% report symptoms worsening during pregnancy (50), while subsets of patients in other small studies report symptom improvement (51, 52). Similar to ME/CFS, 9% of POTS patients (*n* = 1,933) in a cross-sectional survey study report that pregnancy triggered their POTS (35).

### 3.3. Ehlers-Danlos syndrome (EDS), connective tissue disorders (CTDs), and reproductive health

Approximately 23% of LC patients may fit within a musculoskeletal and nervous system phenotype characterized in part by having a higher prevalence of connective tissue disorders (CTDs), according to an EHR study of over 34,000 LC patients (53). The US Centers for Disease Control and Prevention identified systemic involvement of connective tissue as a COVID-19-related condition (54). While the prevalence of CTDs in LC is not yet established, case studies suggest that some patients develop joint hypermobility post-COVID-19 (55).

CTDs, including EDS and its largest subtype hypermobile EDS (hEDS) (56), are relatively common in patients with ME/CFS and POTS (57, 58). hEDS symptoms include joint subluxations, stretchy skin, pain, and fatigue, as well as cardiovascular, gastrointestinal, neurological, and musculoskeletal manifestations, including spinal conditions (56). Exact prevalence varies across studies, but some have found that up to 31% of POTS patients (*n* = 91) (57) and 20% of ME/CFS patients have hEDS (*n* = 229) (58), while 50%-81% of ME/CFS patients are hypermobile (*n* = 229, and *n* = 63) (58, 59) — striking considering the estimated general population prevalence of EDS is 0.2%–3.4% and hypermobility is 12–28% (56). Diagnosis can take years (56) and hEDS has strict diagnostic criteria, so the overall prevalence of CTDs in these illnesses may be higher than these hEDS statistics suggest. While mechanisms of CTDs are under-researched, especially in relation to infection-associated chronic illness, some researchers suspect that mast cell activation (MCA) may be implicated in some CTDs because mast cell mediators like tryptase and histamine can damage collagen (60, 61). Notably, symptoms of MCA are prevalent in LC patients (62), and there are substantial rates of diagnostic comorbidity between mast cell activation syndrome (MCAS) and EDS (63), ME/CFS (64), and POTS (65).

Menstrual symptoms can be severe in EDS patients: 50%–76% (*n* = 26, *n* = 386, *n* = 1,352) report menorrhagia (heavy periods) (66–68); 72% (*n* = 386) report dysmenorrhea (menstrual cramps) (67), 40% (*n* = 1,352) severe dysmenorrhea (68); 16% (*n* = 1,352) experience related moderate to severe pain (68); and 31% (*n* = 1,352) have intermenstrual bleeding (68) (studies lack controls). Furthermore, 43%–64% (*n* = 386, and *n* = 1,146) of EDS patients report dyspareunia (painful intercourse) (67, 69), and 50% (*n* = 1,146) report vulvodynia (pain around the vagina) (69).

Serious pregnancy complications are prevalent in patients with EDS and CTDs, but less than half are informed about these risks (68). In a 16-year population-based retrospective study, pregnancies in EDS patients were associated with higher rates of adverse outcomes, including prematurity, cervical incompetence, antepartum hemorrhage, placenta previa, delivery by cesarean section, longer postpartum stays, maternal death, and having infants with intra-uterine growth restriction (70). In a survey study of 1,352 women with EDS without controls, 43.3% experienced infertility; 54.5% of those who had been pregnant experienced at least one miscarriage; and 29.6% of those who had given birth had at least one premature birth (68). Multiple studies have found that, compared with the general population, women with the three most common types of EDS have higher rates of spontaneous abortions, miscarriages, stillbirths, and preterm premature rupture of membranes (PPROM) (71).

### 3.4. Endometriosis

Endometriosis patients may have an increased risk of developing LC (aHR = 1.19, 95% CI 1.11–1.28) based on a population-based retrospective cohort-matched study using data from electronic health records of non-hospitalized LC patients; however, more research is needed to understand contributing factors (72). Endometriosis is a chronic, systemic disease where tissue similar to the lining of the uterus grows outside the uterus (73, 74). The disease affects 10% of reproductive-age girls and women (75), as well as premenarcheal girls (76) and postmenopausal women (77), nonbinary and transgender people, and, in rare cases, men (78–80). Endometriosis can be associated with a range of severe, disabling symptoms, including but not limited to pain with menstruation, penetration, bowel movement, and/or urination, chronic pain, infertility, fatigue (75), and with occurrences like preeclampsia and other adverse pregnancy outcomes (81, 82). Invasive surgical and histological diagnosis, which is widely considered the gold standard to diagnose (83), stigma, symptom normalization, and lack of practitioner awareness (75) drive average diagnostic delays of up to 11 years (84–87), which may reduce understanding of endometriosis prevalence in LC.

Approximately 36% of women with ME/CFS (*n* = 36) and 20% of women with POTS (*n* = 65) report endometriosis (42, 48, 88). Reduced natural killer cell cytotoxic function (89), macrophage alterations (90), lowered cortisol (91), elevated oxidative stress (92), and allergies (93) are implicated in endometriosis, as well as in ME/CFS (94–96), highlighting overlaps and the role of dysfunctional immune and endocrine systems in both diseases. Some of these mechanisms may also play a role in LC (2, 97–99).

Despite the prevalence of and disablement caused by endometriosis, its pathogenesis remains unknown (100). A lesser known and under-investigated hypothesis proposes that microbes may contribute to endometriosis etiopathology (i.e., “bacterial contamination hypothesis”) (101) *via* an inflammatory response and TLR4 (102–104) and TLR2 activation (104, 105). This research is notable in the context of studies on pathogen persistence and pathobionts in infection-associated chronic illnesses and how they may be implicated in multiple pathologies (106–108). Studies have found evidence of persistent SARS-CoV-2 infection, antigen reservoirs, and oral and intestinal dysbiosis in subsets of LC patients, which have been linked to gastrointestinal symptoms and an elevated risk of developing LC (5, 6, 109–112).

## 4. Discussion

Research on RH in LC is severely lacking, despite patient-reported symptoms and documented impacts in LC and associated conditions. Studies mentioned in this review across all illnesses have largely been underpowered cross-sectional studies, case reports, and lacking in healthy or other relevant control groups—in part due to historic underfunding of ME/CFS and associated illnesses (113). RH conditions in these illnesses have been under-researched, as has female RH more broadly (14–16), and more research funding should be allocated to study RH within LC and associated illnesses.

**Figure 2:**
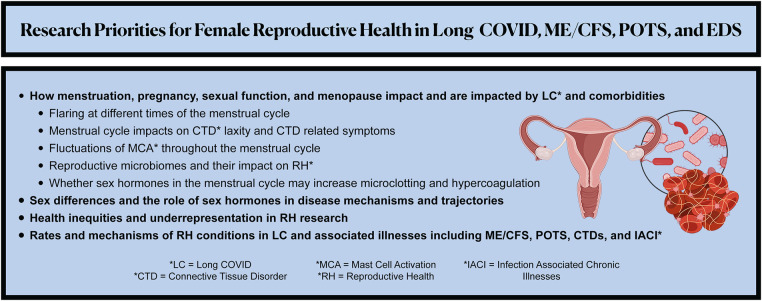
Summarizes the recommended patient-led research priorities to advance the understanding, management, and therapeutics of female reproductive health in Long COVID, myalgic encephalomyelitis/chronic fatigue syndrome (ME/CFS), postural orthostatic tachycardia syndrome (POTS), Ehlers-Danlos Syndrome (EDS) and other associated illnesses.

We urge researchers to investigate the below RH research priorities and to design studies that account for overlapping associated illnesses (Figure 2). Because risk factors for developing LC may include female sex, socioeconomic deprivation, and racial/ethnic minority identity (72), these questions would ideally be explored through large, control-matched prospective longitudinal cohort studies and clinical trials that have significant representation of cisgender women and gender diverse people in all reproductive life phases.

***Sex Differences.*** Female sex is a risk factor for associated illnesses: 70%–80% of patients with ME/CFS, POTS, and EDS are female (10–12). Thus, the roles of chromosomal, hormonal, and anatomical sex are vital to uncovering an understanding of how these illnesses predominate in female patients, worsen with the menstrual cycle and menopause, and could be impacted by hormonal therapies. Given that cisgender females and transgender people may have a higher prevalence of LC than cisgender males (9), LC research should examine LC sex dimorphism, sex differences in immune dysregulation and immune responses to infection, and the role of sex hormones in disease trajectories and RH etiopathology.

***Menstrual Cycle.*** Research is urgently needed to understand how LC symptoms are impacted by the menstrual cycle and to identify therapeutics. It is critical to investigate how the menstrual cycle can drive symptom exacerbation, specifically:

(1) Mechanisms of flares at different times of the menstrual cycles (e.g., whether menstruation can induce PEM).
(2) Whether menstruation and female sex hormones (and possibly synthetic contraceptives), increase hypercoagulation in LC and ME/CFS. Fibrinaloid microclots were found in 100% of LC patients (*n* = 70) (114) and also found in ME/CFS patients (*n* = 25) (115). They are suspected of contributing to pathogenesis, hypoperfusion, and vascular dysfunction (7, 114). Interestingly, sex hormones including estrogen and progesterone have fibrinogen hypercoagulable properties (116). Thus, we encourage research on whether sex hormones impact hypercoagulation and microclotting in LC and ME/CFS.
(3) The link among ACE2 expression, viral persistence, and RH. ACE2 expression in the reproductive tract fluctuates throughout the menstrual cycle (117, 126), and viral persistence is one of the hypothesized mechanisms contributing to the pathophysiology of LC (6, 118).
(4) Whether menstrual cycle-related fluctuations in connective tissue laxity are exacerbated in LC patients with CTDs, as female joint and connective tissue laxity may fluctuate with hormones throughout the menstrual cycle, especially estradiol (119).
(5) Fluctuations of MCA throughout the menstrual cycle (120) and the role of chronic MCA, especially infection-induced chronic MCA, in RH conditions (e.g., MCA may be implicated in the pathophysiology and symptomatology of endometriosis (93, 121, 122) as well as in preeclampsia (123)).
(6) Vaginal and menstrual effluent microbiomes in LC and their impact on RH. Microbiome dysbiosis is a proposed etiopathology of LC (5, 6, 111), and microbiome signatures drive and are driven by gynecological conditions (124–129).
(7) How symptoms impact and are impacted by menopause, given evidence of early menopause (41) and exacerbated symptoms in perimenopausal and postmenopausal women (38).

***Pregnancy*.** It is critical to study how pregnancy impacts and is impacted by LC, especially given that some ME/CFS and POTS patients report symptom improvement or remission during and after pregnancy, while others report symptom worsening or new illness onset with pregnancy (35, 37, 51). Additionally, as research evolves, clinicians working with LC patients who are pregnant or considering pregnancy should be aware of and discuss potential pregnancy-related risks, especially for patients with CTDs.

***Screening for Long COVID Comorbidities and Associated Illnesses.*** Rehabilitation care and research on LC patients should include screening for RH symptoms and conditions (e.g., endometriosis), as well as overlapping illnesses like ME/CFS, POTS, CTDs, and others.

***Additional Reproductive Health Impacts.*** To the best of our knowledge, there are no published studies investigating premenstrual dysphoric disorder (PMDD) and/or female sexual dysfunction following COVID-19 infection [compared to 60 studies on erectile dysfunction post-COVID-19 infection (130)]. It will also be critical to investigate whether pathologies associated with LC increase the risk of cancer in the reproductive tract through mechanisms such as infection-induced dysbiosis, chronic inflammation, and promotion of oncogenic pathways (106, 131).

***Infection-Associated Chronic Illness Beyond Long COVID.*** Lack of discussion related to other infection-associated chronic illnesses and vaccine-onset illness is not due to their lack of relevance but rather absence of research on their RH impacts. Including these patient populations (e.g., chronic Lyme disease) in future research would be beneficial in understanding infection-associated chronic illnesses across antigen triggers.

***Health Inequities in Research.*** The literature reviewed studied largely white populations in high-income countries where these illnesses may be systemically underdiagnosed among people from marginalized groups (132, 133). Clinical research has historically lacked appropriate racial/ethnic/gender representation due to barriers to participation and systemic and institutional racism in healthcare (15, 134). To promote health equity and counteract historic racism and bias in health research, studies must prioritize recruiting and retaining representative samples. Moreover, it will be important for studies to focus on RH experiences in the LC population living in low- and middle-income countries (LMIC), as the frequency and impact of menstrual disorders was higher among adults and adolescents in these countries even before the COVID-19 pandemic (14, 135).

Lack of research and insufficient research funding for RH conditions and infection-associated chronic illnesses contributes to clinical care shortcomings. Gender and racial/ethnic healthcare disparities amplify these shortcomings (136, 137), along with over-psychologicalization of pain and illness in female patients and patients of color (138, 139). Menstrual and sexual health continue to be stigmatized in healthcare (75, 140, 141), a setting where hyperfocus on reproduction in a historically male-dominated OBGYN field has overshadowed women's health and well-being outside of their ability to reproduce (140, 142). Patients with LC and related conditions report often feeling dismissed by clinicians (143), with many of the conditions in this review having extensive diagnostic delays and high rates of initial misdiagnosis (35, 56, 144, 145).

The number of patients with LC continues to rise as COVID-19 persists, yet a paucity of research exists on the female RH implications of LC. LC may be associated with disruptions to the menstrual cycle, gonadal function, ovarian insufficiency, premature menopause, and fertility problems. RH conditions connected to associated illnesses (e.g., ME/CFS, POTS, EDS) include dysmenorrhea, amenorrhea, oligomenorrhea, dyspareunia, endometriosis, infertility, vulvodynia, intermenstrual bleeding, ovarian cysts, uterine fibroids and bleeding, pelvic congestion syndrome, and adverse pregnancy complications, such as maternal mortality, preeclampsia, and premature birth. RH conditions negatively impact quality of life and hinder many women, girls, nonbinary, and trans people from fully participating in the economy and society. We recommend that researchers, patients, and clinicians collaboratively shape a better future for research on the RH impacts of LC and associated illnesses.
